# Supplementary material for: Pseudomonas aeruginosa lectin LecB impairs keratinocyte fitness by abrogating growth factor signalling
Source: Life Sci Alliance. 2019 Nov 15;2(6):e201900422. doi: 10.26508/lsa.201900422 (PMC6858607; doi:10.26508/lsa.201900422)
Supplement: Supplementary file 1 [file LSA-2019-00422_TableS1.doc]

| **-LogP** | **Difference**  **(untreated - treated)** | **Protein** | **Gene name** |
| --- | --- | --- | --- |
| 4.96 | -8.06 | Cation-independent mannose-6-phosphate receptor | IGF2R |
| 3.70 | -7.95 | Insulin-like growth factor 1 receptor | IGF1R |
| 3.52 | -6.94 | Hepatocyte growth factor receptor | MET |
| 5.08 | -3.81 | Epidermal growth factor receptor | EGFR |

**Table S1.** List of coprecipitated growth factor receptors identified by mass spectrometry analysis
