# Supplementary material for: Pseudomonas aeruginosa lectin LecB impairs keratinocyte fitness by abrogating growth factor signalling
Source: Life Sci Alliance. 2019 Nov 15;2(6):e201900422. doi: 10.26508/lsa.201900422 (PMC6858607; doi:10.26508/lsa.201900422)
Supplement: Supplementary file 4 [file LSA-2019-00422_TableS2.doc]

**Table S2.** List of primary antibodies used

| **target** | **species** | **supplier** | **catalog number** | **dilution (application)** |
| --- | --- | --- | --- | --- |
| P-AMPK | rabbit | Cell signaling | 2535 | 1:1000 (WB) |
| AMPK | rabbit | Cell signaling | 2532 | 1:500 (WB) |
| ATG13 | rabbit | Cell signaling | 13468 | 1:1000 (WB) |
| β-actin | mouse | Sigma Aldrich | A5316 | 1:2000 (WB) |
| Cyclin D1 | rabbit | Cell signaling | 2978 | 1:1000 (WB) |
| EEA1 | rabbit | Cell signaling | 3288 | 1:100 (IF) |
| EGFR | rabbit | Santa Cruz | Sc-03 | 1:500 (WB) |
| GAPDH | rabbit | Sigma Aldrich | G9545 | 1:2000 (WB) |
| IGF-1R | rabbit | Cell signaling | 9750 | 1:100 (IF)  1:1000 (WB) |
| IGF-1R | mouse | Abcam | ab16890 | 1:100 (IF, surface staining) |
| IGF-1R | mouse | Invitrogen | AHR0321 | IP  (2 μg/mg lysate) |
| P-IGF-1R (Y1331) | rabbit | Cell signaling | 3021 | 1:1000 (WB) |
| P-IGF-1R (Y1335/1336) | rabbit | Cell signaling | 3024 | 1:1000 (WB) |
| LAMP-1 | rabbit | Cell signaling | 9091 | 1:100 (IF) |
| LC3b | rabbit | Abcam | ab48394 | 1:100 (IF)  1:1000 (WB) |
| LecB | rabbit | Eurogentec | own  production | 1:2000 (WB)  1:50 (IF) |
| P-p44/42 MAPK (ERK1/2) | rabbit | Cell signaling | 4370 | 1:1000 (WB) |
| p44/42 MAPK (ERK1/2) | rabbit | Cell signaling | 4965 | 1:1000 (WB) |
| P-mTOR | rabbit | Cell signaling | 2983 | 1:1000 (WB) |
| mTOR | rabbit | Cell signaling | 5536 | 1:1000 (WB) |
| P. aeruginosa | rabbit | Abcam | ab68538 | 1:50 (IF) |
| RAB9A | rabbit | Cell signaling | 5118 | 1:100 (IF) |
| RAB11 | rabbit | Cell signaling | 5589 | 1:100 (IF) |
| Transferring receptor | mouse | Invitrogen | 13-6800 | 1:200 (IF) |
| α/β-tubulin | mouse | Cell signaling | 2148 | 1:2000 (WB) |
| ubiquitin | mouse | Cell signaling | 3936 | 1:3000 (WB) |
| P-ULK1 (S757) | rabbit | Cell signaling | 14202 | 1:1000 (WB) |
| P-ULK1 (S555) | rabbit | Cell signaling | 5869 | 1:1000 (WB) |
