# Supplementary material for: Pseudomonas aeruginosa lectin LecB impairs keratinocyte fitness by abrogating growth factor signalling
Source: Life Sci Alliance. 2019 Nov 15;2(6):e201900422. doi: 10.26508/lsa.201900422 (PMC6858607; doi:10.26508/lsa.201900422)
Supplement: Supplementary file 5 [file LSA-2019-00422_TableS3.doc]

**Table S3.** List of secondary antibodies used

| target | tag | supplier | catalog number | dilution (application) |
| --- | --- | --- | --- | --- |
| anti-mouse | HRP | Cell signaling | 7076 | 1:1000/1:2000 (WB) |
| anti-rabbit | HRP | Cell signaling | 7074 | 1:1000/1:2000 (WB) |
| anti-mouse | Cy3 | Jackson Immunoresearch | 715-166-150 | 1:200 (IF) |
| anti-rabbit | Cy3 | Jackson Immunoresearch | 711-166-152 | 1:200 (IF) |
| anti-mouse | DyLight 650 | Thermo Fisher | 84545 | 1:200 (IF) |
| anti-rabbit | DyLight 650 | Thermo Fisher | 84546 | 1:200 (IF) |
| anti-rabbit | Alexa Fluor 647 | Thermo Fisher | A-21245 | 1:200 (IF) |
| anti-mouse | DyLight 488 | Thermo Fisher | 35502 | 1:200 (IF) |
